# A Noninvasive Prediction Nomogram for Lymph Node Metastasis of Hepatocellular Carcinoma Based on Serum Long Noncoding RNAs

**DOI:** 10.1155/2019/1710670

**Published:** 2019-07-01

**Authors:** Jie Ma, Li Zhang, Hai-Rong Bian, Zheng-Guo Lu, Lian Zhu, Ping Yang, Zhao-Chong Zeng, Zuo-Lin Xiang

**Affiliations:** ^1^Department of Radiation Oncology, Zhongshan Hospital, Fudan University, Shanghai, China; ^2^Department of Radiation Oncology, Shanghai East Hospital, Tongji University School of Medicine, Shanghai, China

## Abstract

**Background and Objectives:**

Lymph node metastasis (LNM) is common in hepatocellular carcinoma (HCC). In order to intervene HCC LNM in advance, we developed a prediction nomogram based on serum long noncoding RNA (lncRNA).

**Methods:**

Serum samples from 242 HCC patients were gathered and randomly enrolled into the training and validation cohorts. LncRNAs screened out from microarray were quantified with qRT-PCR. Univariate and multivariate analyses were applied for screening independent risk factors. A prediction nomogram was ultimately developed for HCC LNM. The nomogram was estimated by discrimination and calibration tests in the validation cohort. The effects of the candidate lncRNA on the malignant phenotypes of HCC cells were further explored by wound healing assay and colony formation assay.

**Results:**

ENST00000418803, lnc-ZNF35-4:1, lnc-EPS15L1-2:1, BCLC stage, and vascular invasion were selected as components of the nomogram according to the adjusted multivariate analysis. The nomogram effectively predicted the HCC LNM risk among the cohorts with suitable calibration fittings and displayed high discrimination with C-index of 0.89 and 0.85. Moreover, the abnormally high expression of lnc-EPS15L1-2:1 in HCC cell lines showed significant carcinogenic effects.

**Conclusions:**

The noninvasive nomogram may provide more diagnostic basis for treatments of HCC. The biomarkers identified can bring new clues to basic researches.

## 1. Introduction

Liver cancer is the second leading cause of cancer-related death worldwide, with approximately 850, 000 new cases occurring each year [1]. Among all the cases of liver cancer, hepatocellular carcinoma (HCC) accounts for about 90% [2]. Due to the poor prognosis of HCC, its mortality ranks third among all cancer types and shows an upward trend on a global scale [3, 4]. Compared with other prognostic factors, metastasis is one of the main reasons for cancer-related death [5]. Thereinto, lymph node metastasis (LNM) is common within extrahepatic metastases, second only to lung metastasis [6]. It was reported that the intraoperative detection rate of HCC LNM was 0.75%–7.5%, while the detection rate during autopsy was as high as 30.3% [6]. In the previous follow-up, we have found that around 10.3% of HCC patients after hepatectomy will develop LNM [7]. Although external beam radiotherapy is an effective means of treating HCC LNM, the prognosis of patients with LNM is worse than that of patients without LNM [8, 9].

Until now, the mechanisms underlying the development of LNM in HCC are poorly understood. Screening for biomarkers associated with HCC LNM can effectively identify high-risk patients, thereby taking steps to prevent disease progression after curative resection. In the previous studies, we have identified multiple biomarkers that are closely related to HCC LNM, such as hypoxia inducible factor-1*α* (HIF-1*α*), vascular endothelial growth factor (VEGF), and matrix metalloproteinase-2 (MMP-2)[7]. At this stage, we urgently hope to find out noninvasive and specific diagnostic markers to prevent the occurrence of HCC LNM.

Researches have found that lncRNAs are involved in multiple steps of cancer development, and they can be used as sensitive biomarkers to predict cancer metastasis [10, 11]. Moreover, lncRNAs have been identified in body fluids [12–14] and are emerging as novel biomarkers for disease and as targets for disease intervention [15, 16]. Based on the expression profile of serum lncRNAs in HCC LNM [17], we quantitatively analyzed the candidate lncRNAs in the serum samples of 242 patients and evaluated the correlation between the lncRNAs and HCC LNM. The biomarkers with potential predictive efficacy were finally screened out, and a prediction nomogram for HCC LNM based on serum lncRNAs was constructed for the first time in combination with clinicopathological parameters.

By applying prediction nomogram for noninvasive risk assessment, HCC patients at high risk for LNM could receive prophylactic radiotherapy towards regional lymph nodes, which could effectively reduce the incidence of LNM and prolong the overall survival of HCC patients [18–20]. Moreover, the abnormal expression and function of the HCC LNM-related serum lncRNA at cellular level may provide a basis for the mechanism study.

## 2. Materials and Methods

### 2.1. Patients and Serum Samples

**Figure 1:**
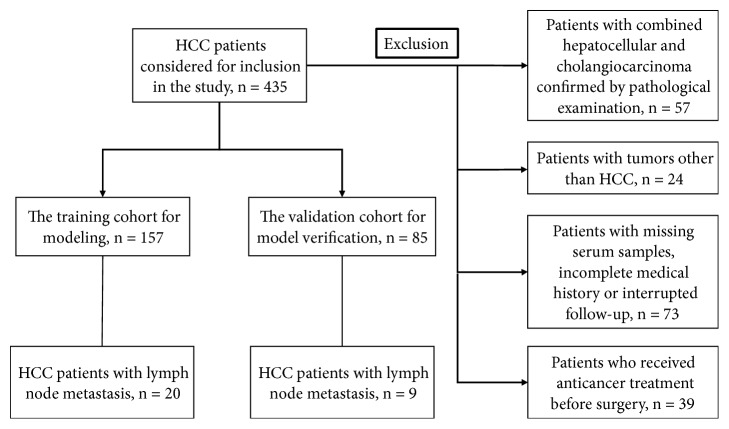
Patients' grouping and exclusion.

The enrolled patients received treatments at Zhongshan Hospital, Fudan University, from 2012 to 2016 and were pathologically diagnosed after surgery. The blood samples from the enrolled patients were collected at the time of surgery and the serums were subsequently extracted with centrifugation (1000×*g*, 10 min, 4°C). The serum samples were than stored at -80°C immediately. All the enrolled patients had complete medical history and clinicopathological data with follow-up and regular clinical examinations and were randomly assigned to the training and validation cohorts in a ratio of 2 : 1. The grouping and exclusion of the enrolled patients are shown in Figure 1. Among the included clinical indicators, the grade of tumor differentiation was determined using the Edmondson grading system and tumor size was measured depending on the maximum diameter of the tumor specimen. The vascular invasion was evaluated with microscopic examination of resected specimens.

All the procedures performed in this study involving human participants were in accordance with the ethical standards of the institutional and national research committee.

### 2.2. Follow-Up and Postoperative Treatments

All the enrolled patients received routine follow-up every 3 months after surgery, which contains clinical examinations and medical history collection. Clinical examinations are composed of abdominal ultrasound examination, liver function test, and blood routine measurement, and all the examinations were performed by doctors who were blind to this study. When HCC LNM was suspected, CT scanning or MRI was carried out immediately for diagnosis. Once diagnosed with HCC LNM, patients were treated with radiotherapy for regional lymph nodes.

### 2.3. RNA Isolation and qRT-PCR

In order to relatively quantify the target lncRNAs, cel-mir-39-3p and GAPDH were used as the external and inner reference, respectively. Total RNAs were isolated from the serum samples using TRIzol LS reagent according to the manufacturer's instructions. Specifically, 0.75 ml TRIzol LS reagent (Life technologies, Carlsbad, CA, US) was added into 0.25 ml serum sample, and 50 fmol of cel-miR-39 was then added for normalization. Cell-derived RNAs were isolated using TRIzol reagent according to the manufacturer's instructions. After assessing the purity of the extracted total RNA, the process of reverse transcription was performed using Prime Script RT reagent Kit (Takara Bio, Shiga, Japan). The primer sequences were listed in Table 1. QRT-PCR analyses were conducted using SYBR® Premix Ex Taq™ (Takara Bio, Shiga, Japan) in the 7500 Real-Time PCR System (Applied Biosystems). The relative expression levels of the candidate lncRNAs were normalized against reference standards by utilizing 2^−△Ct^ method.

### 2.4. Cell Culture

Human hepatocyte cell line QSG-7701 and human HCC cell lines SMMC-7721, HuH-7, HepG2, MHCC-97H, MHCC-97L, and HCC-LM3 were cultured in DMEM containing 10% fetal bovine serum in a constant temperature incubator at 5% CO_2_, 37°C.

### 2.5. Fluorescence In Situ Hybridization

The fixed cells were infiltrated with citrate buffer at room temperature and placed in the cell punching solution for 10 minutes. The cells were immersed in prehybridized working solution at 42°C in the dark and incubated for 2 hours in a wet box. The sample was washed with 0.2×sodium citrate buffer and the hybridization working fluid containing lncRNA probe was then added for overnight rest. After removing the cover glass, the sample was washed at 37°C with 2×sodium citrate buffer, 0.2×sodium citrate buffer, and PBS-T solution. The nuclei were stained with DAPI for 10 minutes at room temperature and then encapsulated. Finally, the cells were observed and photographed by confocal laser scanning microscopy in a field in which the cell division phase was observed.

### 2.6. Lentivirus Transfection

An appropriate amount of lentivirus suspension (moi=30) was added at a cell density of 2×10^5^ /well and cultured at 5% CO_2_, 37°C. After 48 hours, the expression of fluorescent protein TurboGFP was detected under the microscope and the transfection efficiency was confirmed. The stable transfectant was screened by using 2 *μ*g/ml concentration of puromycin.

### 2.7. Wound Healing Assay

The back side of the 6-well plate was marked with a horizontal line and uniformly plated at a cell density of 5×10^5^ per well. After cellular fusion, the vertical lines perpendicular to the marking lines were made with pipette tip in the cell distribution area. The 6-well plate was then washed three times with PBS buffer to ensure the complete removal of exfoliated cells, and the medium with low concentration of serum (2%) was also added. The treated 6-well plate was cultured in a constant temperature incubator at 5% CO_2_ and 37°C and photographed under a microscope at 0, 24, 48, and 72 hours.

### 2.8. Colony Formation Assay

Four hundred and eight hundred cells were laid in a 6 cm culture dish and cultured in a constant temperature incubator at 5% CO_2_ and 37°C. Two weeks later, visible clones were formed in the culture dish and more than 50 cells were found in most single clonal colonies. After removing the culture medium, PBS was gently washed along the medial wall of the dish three times, and 4% polyformaldehyde was used to fix the cells for 30 minutes. After one hour of continuous dyeing with crystal violet dye solution, rinse was performed along the side wall of the dish until clear dark blue colony spots appeared. The colonies containing more than 50 cells were counted three times in each dish, and the average value was taken as the final clone number. Quantitative analysis was carried out by GraghPad Prism software, and the clone formation rate was equal to the number of colonies/inoculated cells.

### 2.9. Statistical Analysis

By using SPSS 22.0 software (Chicago, IL, USA), GraphPad Prism 6.01 (La Jolla, CA, USA), Image J (National Institutes of Health, NIH), MedCalc Statistical Software 18.2.1 (Ostend, Belgium), and R software (version 3.2.3), all the statistical data were shown from at least three separate experiments. Specifically, the correlations between clinicopathological features and HCC LNM were analyzed by Pearson's correlation test and Fisher's exact test. Univariate analysis and multivariate Cox regression analysis (confounding variables that affect the regression coefficient by more than 10% were adjusted) were performed to determine independent risk factors of HCC LNM. The model was finally determined for nomogram with a backward step-down selection process. Estimates of the cutoff value and the model discriminatory ability were measured using time-depended ROC curves. Moreover, calibration curves originating from Hosmer-Lemeshow (H-L) test were applied for evaluation of the nomogram. The* P *< 0.05 were normally considered statistically significant. Vascular invasion was also included as an independent risk factor with slightly higher* P* value (*P* = 0.055) and appropriate hazard ratio.

## 3. Results

### 3.1. Patients' Background Data

Characteristics of all the enrolled HCC patients (157 in the training cohort and 85 in the validation cohort) used to establish a prediction model are summarized in Table 2. Patients of the training cohort were observed until October 2016, and the median follow-up time was 55 months (range, 9-76 months). For the validation cohort, observation lasted until September 2016 and the median follow-up time was 45 months (range, 10-76 months). During the follow-up time, 20 patients (12.7%) in the training cohort and 9 patients (10.6%) in the validation cohort developed LNM.

### 3.2. Screening of Serum Biomarkers with Microarray

Through previous microarray analyses on serum samples, we have identified 235 lncRNAs that were differentially expressed among LNM group and non-LNM group as reported [17]. Among all the significant differential lncRNAs, five had fold change > 2 and FDR values < 0.05 with high expression abundance in serum samples. Lnc-GALR2-1:1 was downregulated with a FDR value of 0.034; ENST00000418803 was downregulated with a FDR value of 0.037; lnc-ZNF35-4:1 was downregulated with a FDR of 0.042; lnc-CAMKK2-3:2 was upregulated with a FDR of 0.037; and lnc-EPS15L1-2:1 was upregulated with a FDR of 0.037. We therefore conducted qRT-PCR to examine the expression of the candidate lncRNAs in serum samples from the training cohort and further evaluated the ability of these potential biomarkers for predicting HCC LNM.

### 3.3. Analysis of Serum Candidate lncRNAs Expression

**Figure 2:**
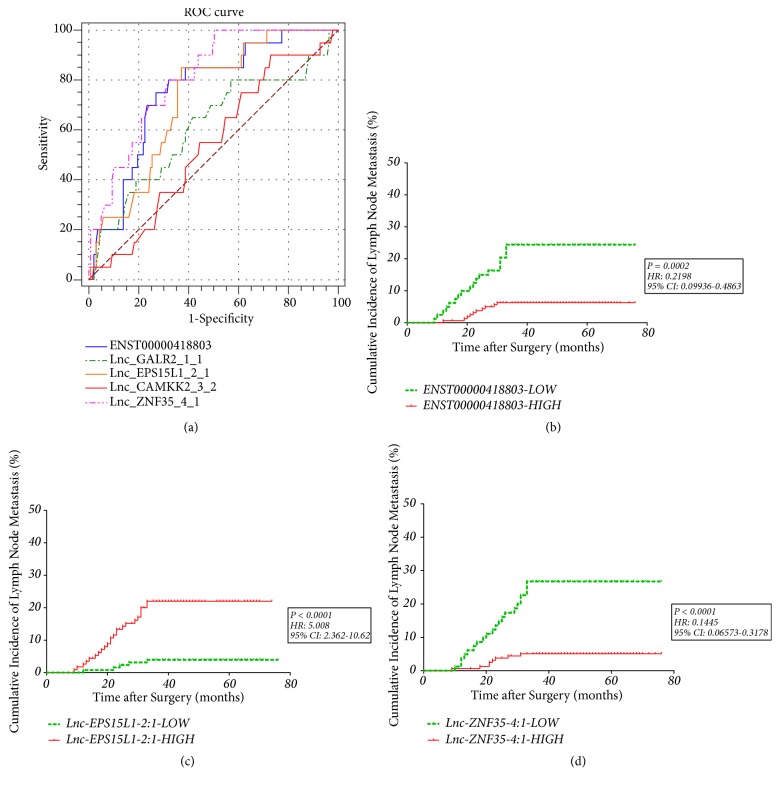
*Screening and analyses of potential serum lncRNAs as biomarkers*. (a) Comparative ROC analysis of the five candidate lncRNAs screened by microarray analyses. (b) Kaplan-Meier and Log-rank analyses on difference in LNM cumulative incidence of patients with low or high expression of ENST00000418803. (c) Kaplan-Meier and Log-rank analyses on difference in LNM cumulative incidence of patients with low or high expression of lnc-EPS15L1-2:1. (d) Kaplan-Meier and Log-rank analyses on difference in LNM cumulative incidence of patients with low or high expression of lnc-ZNF35-4:1.

**Figure 3:**
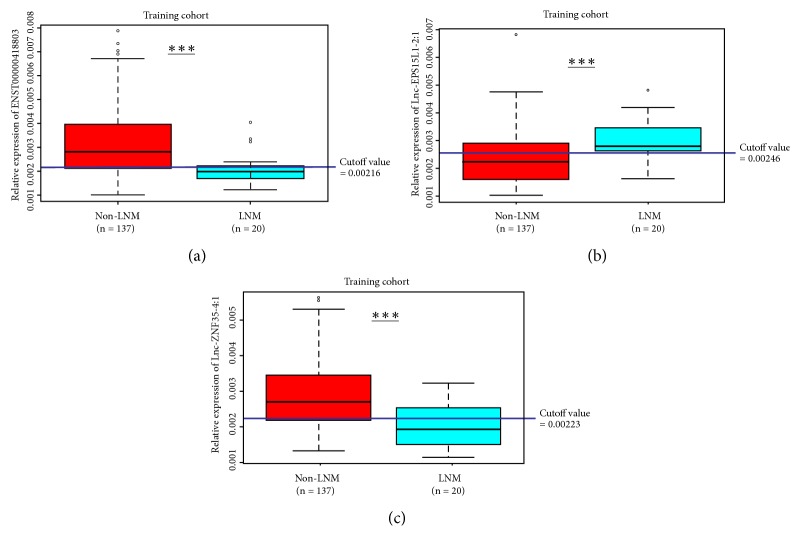
*The optimum cutoff value of the candidate lncRNAs for discrimination of expression levels among the training cohort*. (a) Relative expression of ENST00000418803 was distinguished by cutoff value 0.00216 as low or high expression. (b) Relative expression of lnc-EPS15L1-2:1 was distinguished by cutoff value 0.00246 as low or high expression. (c) Relative expression of lnc-ZNF35-4:1 was distinguished by cutoff value 0.00223 as low or high expression.

With cel-mir-39-3p as an external reference, qRT-PCR was carried out to quantify the relative expression of the candidate lncRNAs in the serum samples. We first assessed the relevance between the serum lncRNA expression and diagnosis of HCC LNM based on the ROC curve and determined the cutoff value for judging lncRNA expression level simultaneously. Relative expressions of lnc-GALR2-1:1 and lnc-CAMKK2-3:2 in serum samples from the enrolled patients were verified to have no statistical significance for predicting HCC LNM. Besides, the remaining three lncRNAs were defined as high- or low-expression level based on the maximum value of Youden's index in the ROC analysis [21]. As showed in Figure 2(a), the AUC of ENST00000418803, lnc-EPS15L1-2:1, and lnc-ZNF35-4:1 were 0.753 (95% CI: 0.678-0.819), 0.721 (95% CI: 0.644-0.790), and 0.766 (95% CI: 0.692-0.830), respectively, which indicated a considerable distinguishing power to HCC LNM. To further determine the diagnostic relevance between the above three lncRNAs and HCC LNM, Kaplan-Meier and Log-rank tests were performed among all patients from the cohorts as Figures 2(b), 2(c), and 2(d) show. The distinctions between non-LNM and LNM groups in the training cohort divided by the optimum cutoff values were displayed in Figure 3. The low expression of ENST00000418803 and lnc-ZNF35-4:1 and the high expression of lnc-EPS15L1-2:1 showed significant correlations with the occurrence of LNM.

In the training cohort of 157 patients, low ENST00000418803 expression was found in 51 of 157 patients (32.5%), high lnc-EPS15L1-2:1 expression in 68 (43.3%), and low lnc-ZNF35-4:1 expression in 55 (35.0%). In the validation cohort, low ENST00000418803 expression was found in 30 of 85 patients (35.3%), high lnc-EPS15L1-2:1 expression in 33 (38.8%), and low lnc-ZNF35-4:1 expression in 27 (31.8%).

### 3.4. Significant Predictors of Lymph Node Metastasis in Hepatocellular Carcinoma

For the training cohort, 19 clinicopathological features consisted of age, gender, HCV-Ab, HBsAg, a-fetoprotein, tumor differentiation, Child-Pugh score, intrahepatic metastasis, tumor size, vascular invasion, BCLC staging, ALT, *γ*-GT, liver cirrhosis, tumor number, and distant metastasis and the expression of the serum lncRNAs mentioned above was considered for the univariate analysis with Cox proportional hazards regression. The associations of the included variables with HCC LNM in the training cohort are summarized in Table 3. Through univariate analysis, vascular invasion (*P *= 0.010), BCLC stage (*P *= 0.023), ENST00000418803 (*P *< 0.001), lnc-EPS15L1-2:1 (*P *< 0.001), and lnc-ZNF35-4:1 (*P *= 0.001) were selected to be significantly associated with LNM in HCC patients, whereas age (*P *= 0.286), gender (*P *= 0.187), HBsAg (*P *= 0.248), HCV-Ab (*P *= 0.997), a-fetoprotein (*P *= 0.539), tumor differentiation (*P *= 0.611), Child-Pugh score (*P *= 0.998), intrahepatic metastasis (*P *= 0.365), tumor size (*P *= 0.509), alanine aminotransferase (ALT) (*P *= 0.456), *γ*‐glutamyltransferase (*γ*-GT) (*P *= 0.878), liver cirrhosis (*P *= 0.756), tumor number (*P *= 0.585), and distant metastasis (*P *= 0.752) displayed no significant association with LNM. Statistically significant variables were further adopted for multivariate analysis. By adjusted multivariate analysis, the following five variables were found to be independent risk factors for LNM in HCC: vascular invasion (*P* = 0.055, HR: 2.5, 95% CI: 1.0 ~ 6.5), BCLC stage (*P *= 0.007, HR: 4.2, 95% CI: 1.5 ~ 12.0), ENST00000418803 (*P *< 0.001, HR: 0.2, 95% CI: 0.1 ~ 0.5), lnc-EPS15L1-2:1 (*P *= 0.001, HR: 8.7, 95% CI: 2.5 ~ 30.6), and lnc-ZNF35-4:1 (*P *= 0.025, HR: 0.3, 95% CI: 0.1 ~ 0.9).

### 3.5. Construction of Prediction Nomogram for LNM in HCC

**Figure 4:**
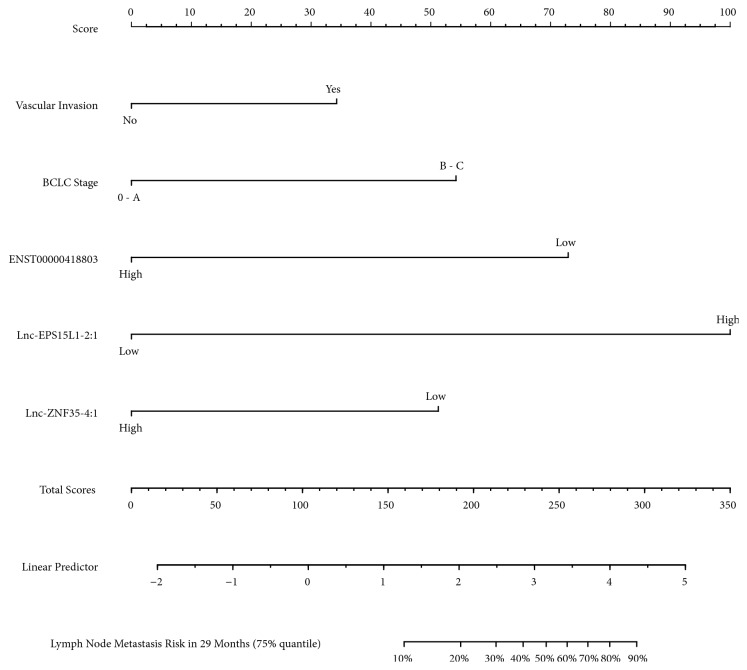
The visible nomogram based on lncRNAs and clinical indexes for predicting LNM in postoperative HCC patients.

**Figure 5:**
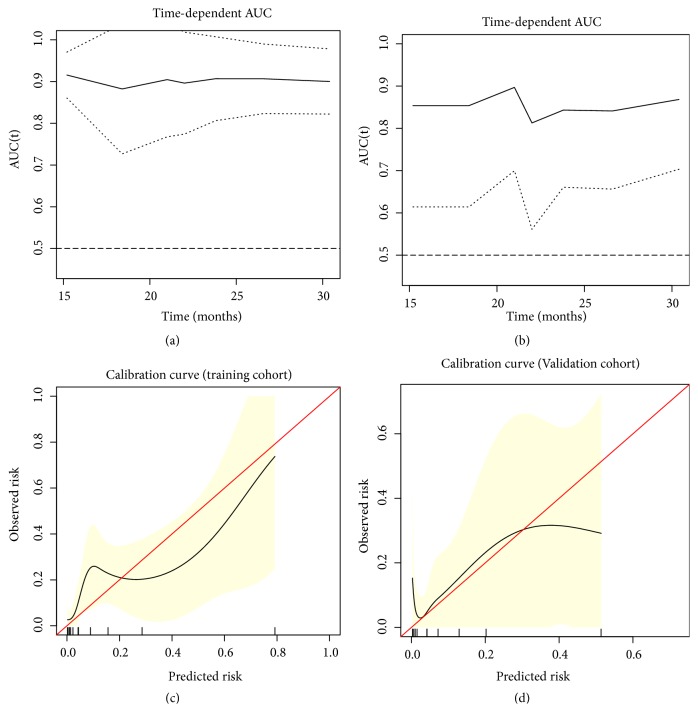
*Time-dependent AUC and calibration tests of the nomogram among the training and validation cohorts*. (a) Time-dependent AUC of the nomogram among the training cohort. (b) Time-dependent AUC of the nomogram among the validation cohort. (c) Calibration curve of the nomogram among the training cohort. (d) Calibration curve of the nomogram among the validation cohort.

As shown in Figure 4, the following five independent risk variables from multivariate cox regression analyses were selected into the visible nomogram to predict the risk of LNM: high lnc-EPS15L1-2:1 expression has the highest score of 100; low ENST00000418803 has the score of 73; low lnc-ZNF35-4:1 has the score of 51; BCLC stage; and vascular invasion was scored as 54 and 34, respectively. Considering the time distribution of LNM occurrence and the time-dependent AUC in the both cohorts, we determined the 29 months' time (75% quartile) as an observation point, in which the nomogram has the best prediction performance and application value. The sum of score from all the included risk factors can further correspond to the risk assessment of LNM occurring within 29 months. The prediction nomogram demonstrated a good accuracy with stable and favourable time-dependent AUC among the training cohorts (Figure 5(a)). Calibration curves revealed a suitable calibration between the predictive LNM risk and the observed LNM risk as well (Figure 5(c)). Harrell's C-index of the stepwise selected model for LNM prediction was 0.89, which indicated a sound discrimination ability.

### 3.6. Validation for the Predictive Value of the lncRNA-Based Nomogram

As Figure 5(b) shows, the trend of AUC was stable and satisfied in the interval of 15 to 31 months' observation time point, which revealed a good prediction performance for LNM within the optimum time (29 months' time). The calibration curve noted that the nomogram was well calibrated with a favorable fitting between the observed and predicted risk (Figure 5(d)). Furthermore, Harrell's C-index 0.85 of the model demonstrated good discrimination in the validation step.

### 3.7. Role of Lnc-EPS15L1-2:1 in Lymph Node Metastasis of HCC Cells

The aforementioned analyses showed that the high expression of serum lnc-EPS15L1-2:1 had a strong correlation with the occurrence of lymph node metastasis in HCC patients. However, the source of free lnc-EPS15L1-2:1 and the biological functions associated with HCC LNM remain unknown.

**Figure 6:**
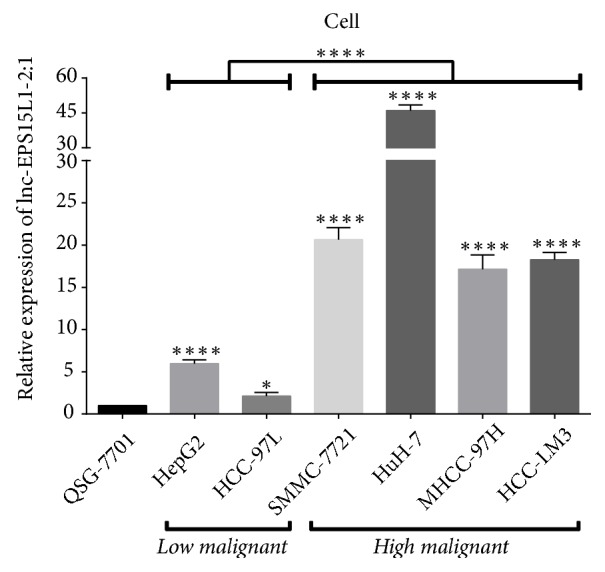
*Detection and analysis of lnc-EPS15L1-2:1 expression at the cellular level*. Lnc-EPS15L1-2:1 expression was abnormally elevated in HCC cells and was associated with high malignancy of HCC cells (*∗*:* P* < 0.05; *∗∗∗∗*:* P* < 0.0001).

**Figure 7:**
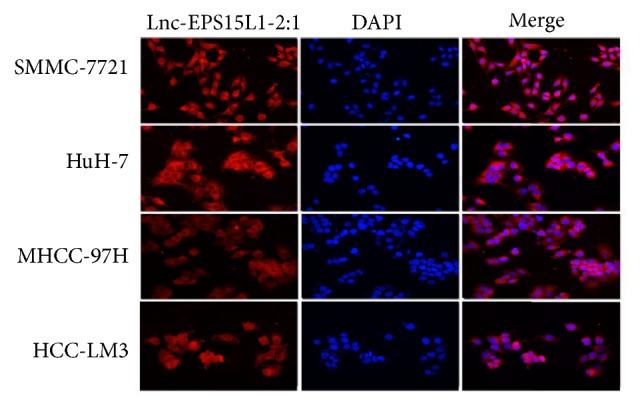
*Determination of subcellular distribution of lnc-EPS15L1-2:1 using FISH*. Confocal images showed that most of lnc-EPS15L1-2:1 are distributed in the cytoplasm of HCC cells.

We performed qRT-PCR on the expression of lnc-EPS15L1-2:1 in human hepatocyte line QSG-7701 and human HCC cell lines SMMC-7721, Huh-7, HepG2, MHCC-97H, MHCC-97L, and HCC-LM3. Among these, SMMC-7721, HuH-7, MHCC-97H, and HCC-LM3 cell lines have high invasion and metastasis characteristics, which are highly malignant. On the contrary, HepG2 and MHCC-97L cell lines show low malignancy in invasion and metastasis. As shown in Figure 6, the expression of lnc-EPS15L1-2:1 in HCC cells was higher than that in normal hepatocytes and was significantly elevated in highly malignant cell lines. Fluorescence in situ hybridization (FISH) further confirmed the subcellular distribution of lnc-EPS15L1-2:1 which was mainly cytoplasmic (Figure 7).

**Figure 8:**
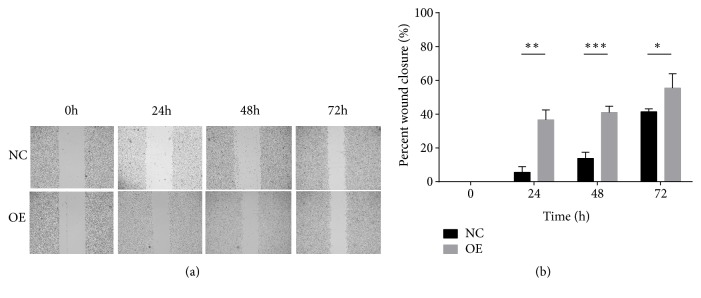
*Wound healing assay for SMMC-7721 cell line*. Comparison of migration degrees of HCC cells in the overexpression group (OE) and the control group (NC) at 0, 24, 48, and 72 hours (*∗*:* P *< 0.05; *∗∗*:* P* < 0.01; *∗∗∗*:* P* < 0.001).

**Figure 9:**
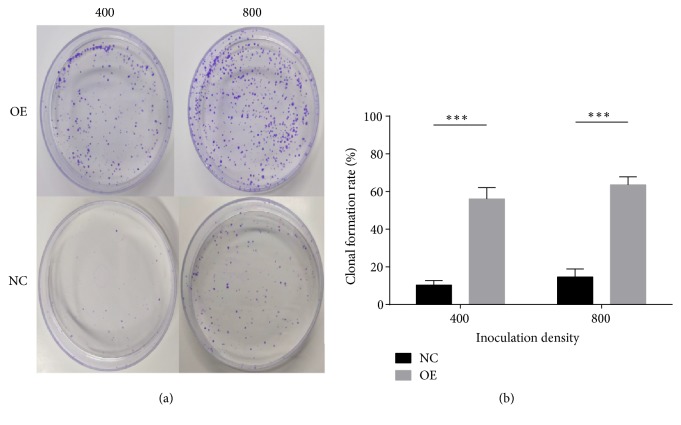
*Colony formation assay for SMMC-7721 cell line.* Comparison of clonogenicity degrees of HCC cells in the overexpression group (OE) and the control group (NC) at 400 and 800 inoculation densities (*∗∗∗*:* P* < 0.001).

The formation of tumor metastases depends on both high invasion and migration potential and strong clone-forming ability. Therefore, we performed wound healing assay and colony formation assay after the overexpression of SMMC-7721 cell line by lentiviral transfection. As shown in Figures 8 and 9, lnc-EPS15L1-2:1 significantly enhanced the migration and clonality of HCC cells.

## 4. Discussion

Hepatocellular carcinoma (HCC) is one of the most common malignant tumors worldwide. The incidence of LNM in extrahepatic metastases of HCC is approximately 33.8%, and the overall survival of untreated HCC patients with LNM is only about three months [6, 22]. Lymph node metastasis is a clear prognostic factor in treatments of cancer and has a significant impact on long-term survival of patients [23]. However, imaging technique is still not sensitive to the initial diagnosis of lymph node micrometastasis. It is necessary to build a specific model that predicts LNM risk in HCC patients for preventive intervention.

In general, the free nucleic acid in the circulation of tumor patients is mainly derived from the frequent apoptosis and necrosis of cells [24, 25]. Therefore, the abnormal expression of some cell-free RNA can partly reflect the expression profile of cancer cells, which is significantly related to the malignant process of cancer [26]. Aberrant expressions of lncRNAs have been reported to participate in diverse biological processes in cancer, including LNM [27–29]. Furthermore, lncRNAs are becoming a type of potential biomarkers for disease prediction [12, 30]. In the current study, we therefore sought to identify sensitive serum lncRNAs as noninvasive biomarkers to predict LNM in HCC.

We constructed and then validated a novel prediction nomogram based on three lncRNAs to predict LNM for HCC patients after hepatectomy. Our lncRNA-based nomogram incorporates the following five independent prognostic factors: BCLC stage, vascular invasion, low expression of ENST00000418803 and lnc-ZNF35-4:1, and high expression of lnc-EPS15L1-2:1. Using a linear predictor of 1.7 as the optimum cutoff value, 18.5% (n = 29) patients in the training cohort are identified as high-risk group, and 48.3% (n = 14) patients in the high-risk group developed LNM within the follow-up period. When applying the nomogram to the verification step, 14.1% (n = 12) patients in the validation group were allocated to the high-risk group, and 41.7% (n = 5) of whom were diagnosed with LNM. To our knowledge, this is the first report of serum lncRNA-based prediction nomogram in HCC, especially with respect to LNM. Utilizing this nomogram, posthepatectomy HCC patients can be accurately classified into groups with low and high risk of LNM at the early stage. The nomogram could serve as a valuable criterion for determining optimal treatment strategies for HCC patients.

We have two conjectures about the source of the lncRNAs that have potential predictive effects on HCC LNM. One possibility is that the abnormal cell-free lncRNAs may originate from the transformation process of premetastatic niches mediated by circulating tumor cells or tumor exosomes. NONCODE database displayed the notion that lnc-EPS15L1-2:1 are significantly highly expressed in lymph node compared with other tissues [31]. Another possibility is that changes in the expression profile of lncRNAs in tumor cells promote metastatic propensity. Cis analyses of protein-coding genes adjacent to the lncRNAs loci uncovered their possible roles in tumor malignant phenotype. Specifically, Kruppel-like factor 2 (KLF2), associated gene of lnc-EPS15L1-2:1 from Cis and Trans analyses, was reported as a terminal component of tumor proliferation and metastasis related pathway axis [32–34]. And ENST00000418803 associated gene Sad1 and UNC84 domain containing 2 (SUN2) act as novel suppressors in cancer [35, 36].

There is a strong correlation between the high expression of serum lnc-EPS15L1-2:1 and the occurrence of HCC LNM. Our further experiments confirmed that lnc-EPS15L1-2:1 is highly expressed in HCC cells compared with normal hepatocytes and is associated with the malignancy of HCC cells. Moreover, lnc-EPS15L1-2:1, mainly distributed in the cytoplasm, significantly promotes the migration and clonality of HCC cells. We are about to investigate the specific role of lnc-EPS15L1-2:1 in HCC LNM in the next stage of research.

Some limitations should also be acknowledged as follows. As it was a retrospective cohort study, and because of the limited quantity of patients involved, the results need to be further validated in a large scale of prospective study.

In conclusion, the nomogram based on serum ENST00000418803, lnc-ZNF35-4:1, lnc-EPS15L1-2:1, BCLC stage, and vascular invasion has good predictive performance for HCC LNM, and HCC patients at high risk of LNM may benefit from this. Moreover, as an independent risk factor, overexpression of lnc-EPS15L1-2:1 may mediate the occurrence of HCC LNM at the cellular level, which needs further verification. This study will provide new ideas for the clinical diagnosis and mechanism research of HCC LNM.

## Figures and Tables

**Table 1:** Primer sequences designed for qRT-PCR.

Gene ID	Forward primer	Reverse primer	Product length	Tm (°C)
lnc-GALR2-1:1	5′-CTGAGCACCAAAAGTCTGGC-3′	5′-GCACTCCCATTGTTCGGGAT-3′	90	60

ENST00000418803	5′-GCATAGGCATAAATTAGGATCTGGAGA-3′	5′-TGGAAGGGAGGGGAGTGGAT-3′	128	60

lnc-ZNF35-4:1	5′-GAGTGAGTTTGTGGACCCAGATTTC-3′	5′-GGTCATGTTATATGCTTGCCTTCAG-3′	111	60

lnc-EPS15L1-2:1	5′-TCCCCAACTTTCTGAGACTGT-3′	5′-AAGGAAGTGTGATCCGAGAGGT-3′	133	60

lnc-CAMKK2-3:2	5′-GGATGCCCTAAAGGACTCCG-3′	5′-CCTCCAGATGGCCGTTTTCT-3′	94	60

**Table 2:** Clinicopathological characteristics of the study cohorts.

Variable	Training cohort				Validation cohort			
	All	NLNM	LNM	*P*	All	NLNM	LNM	*P*
	(n=157)	(n=137)	(n=20)		(n=85)	(n=76)	(n=9)	
Age								
≤ 51	69	58	11	0.339	39	33	6	0.290
> 51	88	79	9		46	43	3	
Gender								
male	131	112	19	0.201	65	58	7	1
female	26	25	1		20	18	2	
HBsAg								
negative	12	9	3	0.183	6	6	0	1
positive	145	128	17		79	70	9	
HCV-Ab								
negative	154	134	20	1	81	75	6	0.003*∗*
positive	3	3	0		4	1	3	
AFP, ng/mL								
≤ 20	44	37	7	0.438	27	25	2	0.712
> 20	113	100	13		58	51	7	
Tumor differentiation								
I-II	118	104	14	0.584	68	61	7	1
III-IV	39	33	6		17	15	2	
Child-Pugh score								
A	152	132	20	1	83	75	8	0.202
B	5	5	0		2	1	1	
Intrahepatic metastasis								
no	114	101	13	0.428	66	61	5	0.108
yes	43	36	7		19	15	4	
Tumor size, cm								
≤ 5	89	79	10	0.630	55	48	7	0.483
> 5	68	58	10		30	28	2	
Vascular invasion								
no	117	107	10	0.012*∗*	59	54	5	0.446
yes	40	30	10		26	22	4	
BCLC stage								
0-A	129	116	13	0.054	74	69	5	0.014*∗*
B-C	28	21	7		11	7	4	
ALT, U/L								
≤ 40	74	63	11	0.481	41	36	5	0.733
> 40	83	74	9		44	40	4	
*γ*-GT, U/L								
≤ 50	35	30	5	0.776	18	16	2	1
> 50	122	107	15		67	60	7	
Liver cirrhosis								
no	18	16	2	1	9	9	0	0.588
yes	139	121	18		76	67	9	
Tumor number								
single	108	93	15	0.613	64	60	4	0.037*∗*
multiple	49	44	5		21	16	5	
Distant metastasis								
no	97	84	13	0.810	55	53	2	0.008*∗*
yes	60	53	7		30	23	7	
ENST00000418803								
low expression	51	37	14	< 0.001*∗*	30	24	6	0.061
high expression	106	100	6		55	52	3	
Lnc-EPS15L1-2:1								
low expression	89	86	3	< 0.001*∗*	52	49	3	0.084
high expression	68	51	17		33	27	6	
Lnc-ZNF35-4:1								
low expression	55	41	14	< 0.001*∗*	27	20	7	0.004*∗*
high expression	102	96	6		58	56	2	

HBsAg: hepatitis B surface antigen; HCV-Ab: hepatitis C virus antibody; AFP: a-fetoprotein; ALT: alanine aminotransferase; *γ*-GT: *γ*-glutamyl transferase; BCLC stage: Barcelona Clinic Liver Cancer stage; *∗*: significance values.

**Table 3:** Univariate and multivariate analyses of factors associated with lymph node metastasis of hepatocellular carcinoma among the training cohort.

Clinicopathological factors	Univariate			Multivariate (Adjusted)		
	Hazard ratio	95% CI	*P*	Hazard ratio	95% CI	*P*
Age						
≤ 51	1.0		0.286	-	-	-
> 51	0.6	0.3~1.5		-	-	
Gender						
male	1.0		0.187	-	-	-
female	3.9	0.5~28.9		-	-	
HBsAg						
negative	1.0		0.248	-	-	-
positive	0.5	0.1~1.7		-	-	
HCV-Ab						
negative	1.0		0.997	-	-	-
positive	0.0	0.0~Inf		-	-	
AFP, ng/mL						
≤ 20	1.0		0.539	-	-	-
> 20	0.7	0.3~1.9		-	-	
Tumor differentiation						
I-II	1.0		0.611	-	-	-
III-IV	1.3	0.5~3.3		-	-	
Child-Pugh score						
A	1.0		0.998	-	-	-
B	0.0	0.0~Inf		-	-	
Intrahepatic metastasis						
no	1.0		0.365	-	-	-
yes	1.5	0.6~3.8		-	-	
Tumor size, cm						
≤ 5	1.0		0.509	-	-	-
> 5	1.3	0.6~3.2		-	-	
Vascular invasion						
no	1.0		0.010*∗*	1.0		0.055
yes	3.2	1.3~7.6		2.5	1.0~6.5	
BCLC stage						
0-A	1.0		0.023*∗*	1.0		0.007*∗*
B-C	2.9	1.2~7.3		4.2	1.5~12.0	
ALT, U/L						
≤ 40	1.0		0.456	-	-	-
> 40	0.7	0.3~1.7		-	-	
*γ*-GT, U/L						
≤ 50	1.0		0.878	-	-	-
> 50	0.9	0.3~2.5		-	-	
Liver cirrhosis						
no	1.0		0.756	-	-	-
yes	1.3	0.3~5.4		-	-	
Tumor number						
single	1.0		0.585	-	-	-
multiple	0.8	0.3~2.1		-	-	
Distant metastasis						
no	1.0		0.752	-	-	-
yes	0.9	0.3~2.2		-	-	
ENST00000418803						
low expression	1.0		< 0.001*∗*	1.0		< 0.001*∗*
high expression	0.2	0.1~0.5		0.2	0.1~0.5	
Lnc-EPS15L1-2:1						
low expression	1.0		< 0.001*∗*	1.0		0.001*∗*
high expression	9	2.6~30.7		8.7	2.5~30.6	
Lnc-ZNF35-4:1						
low expression	1.0		0.001*∗*	1.0		0.025*∗*
high expression	0.2	0.1~0.5		0.3	0.1~0.9	

HBsAg: hepatitis B surface antigen; HCV-Ab: hepatitis C virus antibody; AFP: a-fetoprotein; ALT: alanine aminotransferase; *γ*-GT: *γ*-glutamyl transferase; BCLC stage: Barcelona Clinic Liver Cancer stage; CI, confidence interval; *∗*: significance values.

## Data Availability

The data used to support the findings of this study are available from the corresponding author upon request.

## Abbreviations

HCC: Hepatocellular carcinoma
LNM: Lymph node metastasis
lncRNA: Long noncoding RNA
qRT-PCR: Quantitative reverse transcription polymerase chain reaction
HIF-1*α*: Hypoxia inducible factor-1*α*
VEGF: Vascular endothelial growth factor
MMP2: Matrix metalloproteinase-2
BCLC: Barcelona Clinic Liver Cancer
AFP: Alpha-fetoprotein
ALT: Alanine aminotransferase
*γ*-GT: *γ*-glutamyltransferase
ROC curve: Receiver operating characteristic curve
AUC: Area under the curve.
